# Predictive Risk of Radiation Induced Cerebral Necrosis in Pediatric Brain Cancer Patients after VMAT *Versus* Proton Therapy

**DOI:** 10.3390/cancers7020617

**Published:** 2015-04-13

**Authors:** Derek Freund, Rui Zhang, Mary Sanders, Wayne Newhauser

**Affiliations:** 1 Department of Radiation Oncology, Mary Bird Perkins Cancer Center, 4950 Essen Ln., Baton Rouge, LA 70809, USA; E-Mails: dfreund@wkhs.com (D.F.); msanders@marybird.com (M.S.); wnewhauser@marybird.com (W.N.); 2 Department of Physics and Astronomy, Louisiana State University, Nicholson Hall, Tower Dr., Baton Rouge, LA 70810, USA

**Keywords:** radiation induced cerebral necrosis, brain cancer, volumetric modulated arc therapy, proton therapy

## Abstract

Cancer of the brain and central nervous system (CNS) is the second most common of all pediatric cancers. Treatment of many of these cancers includes radiation therapy of which radiation induced cerebral necrosis (RICN) can be a severe and potentially devastating side effect. Risk factors for RICN include brain volume irradiated, the dose given per fraction and total dose. Thirteen pediatric patients were selected for this study to determine the difference in predicted risk of RICN when treating with volumetric modulated arc therapy (VMAT) compared to passively scattered proton therapy (PSPT) and intensity modulated proton therapy (IMPT). Plans were compared on the basis of dosimetric endpoints in the planned treatment volume (PTV) and brain and a radiobiological endpoint of RICN calculated using the Lyman-Kutcher-Burman probit model. Uncertainty tests were performed to determine if the predicted risk of necrosis was sensitive to positional errors, proton range errors and selection of risk models. Both PSPT and IMPT plans resulted in a significant increase in the maximum dose to the brain, a significant reduction in the total brain volume irradiated to low doses, and a significant lower predicted risk of necrosis compared with the VMAT plans. The findings of this study were upheld by the uncertainty analysis.

## 1. Introduction

Cancer of the brain and central nervous system (CNS) is the second most common of all pediatric cancers. At a rate of 5.1 out of every 100,000 cases per year, its incidence is second only to leukemia [1]. The Surveillance Epidemiology and End Result (SEER) program reported that astrocytomas and other gliomas make up approximately 67% of pediatric brain cancers, and ependymomas make up 9% [2]. Treatment for these tumors often incorporates radiation therapy and/or chemotherapy [3] with typical prescriptions of 50–60 Gy in 1.8–2Gy/fraction to the planned treatment volume (PTV) [4]. Proton therapies have been administered up to 59.4 Gy (RBE) to the PTV which includes the gross tumor volume (GTV) plus 2–5 cm expansion [5].

The potential side effects from CNS irradiation can include acute effects and late effects. Acute affects are seen early and can include alopecia, erythema, otitis, tinnitus, or even temporary demyelination [3]. Alternatively late effects onset more than 6 months after treatment and tend to be more severe, including spinal myelopathy, endocrine and cognitive dysfunction, secondary cancers, and radiation induced necrosis [6]. With approximately 72% and 73% five-year survival rate of patients with primary brain and CNS tumors between the ages 0–14 and 0–19, respectively, the risk of late effects is a major concern [7].

Radiation induced cerebral necrosis (RICN) has a latency of as little as 3 month or as long as 13 year after treatment and can vary in severity from asymptomatic radiographic changes, cognitive dysfunction, stroke to death. [8]. Due in part to a long latency, difficulty of diagnosis, and apparently low incidence, the true incidence of RICN is poorly known [9]. Some reports indicate that depending on the treatment protocol the actual incidence could be anywhere from 3% to 24% [10]. Although the expected incidence of RICN may be low, it is a severe and potentially lethal late effect that can extremely impact these patients quality of life. Little is known about the mechanism by which RICN occurs but it has been suggested by Fink *et al.* [8] that it may be a result of ischemia resulting from vascular endothelial injury or from the loss of or injury to oligodendrocytes. Other potential risk factors include the total dose given, the total volume irradiated, fraction size, concurrent chemotherapy, age at exposure, treatment modality, *etc.* [8,11,12,13,14].

Recent retrospective studies have assessed some of these risk parameters. A recent study on the quantitative analysis of normal tissue effects in the clinic (QUANTEC) looked at the radiation dose-volume effects in the brain [12]. Their result indicates that the dose to cause radiation is high with a predicted 5% and 10% risk of symptomatic necrosis to occur at 72 Gy and 90 Gy given in 2 Gy/fraction increments [12]. Additionally they added that fractions sizes greater than 2 Gy increased the risk. Another recent study by Murphy *et al.* [14] found that the percentage of the infratentorial brain receiving 50, 52 and 54 Gy were significant predictors of risk of necrosis for pediatric patients being treated for craniopharyngioma. Ruben *et al.* [10] found that the risk of necrosis increases with increasing dose, fraction size and the addition of chemotherapy.

All of the above studies were based on patients treated with photon therapy. Boehling *et al.* [15] compared intensity modulated photon therapy (IMRT) with intensity modulated proton therapy (IMPT) for pediatrics with craniopharyngioma and found IMPT spared dose to the cerebral vasculature compared to IMRT. Other studies have found similar effects of normal tissue sparing for pediatric medulloblastoma when comparing passively scattered proton therapy (PSPT) to conventional photon therapy [16].

The primary focus of this investigation is to compare the risk of RICN between volumetric modulated arc therapy (VMAT) and passively scattered proton therapy (PSPT) as well as intensity modulated proton therapy (IMPT). Comprehensive uncertainty analysis was also performed.

## 2. Methods and Materials

A patient database was constructed with 13 anonymized CT data sets from pediatric patients with varying age, sex and treatment sites. These patients were consecutively sampled from patients that were previously treated with proton cranial spinal irradiation (CSI) at the University of Texas at M.D. Anderson between 2007 and 2009. Five of these patients were used for our ependymoma comparison and eight for the astrocytoma (glioma) comparison. A list of these patients based on age, sex and disease type can be seen Table 1.

Contouring was performed in Phillips Pinnacle treatment planning system (TPS). A clinical treatment volume (CTV) was created for each patient by the same board certified radiation oncologist to represent the glioma and ependymoma gross tumor volume (GTV) plus any subclinical disease that may exist. An additional 1 cm margin was then added to the CTV to define the planned treatment volume (PTV). This additional PTV margin accounts for patient setup error. A PTV reduction of no more than 0.5 cm was allowed to account for organs at risk or boney anatomy. Additional beam specific proximal margins (PM) and distal margins (DM) were determined for proton plans due to range uncertainties inherent in proton beam therapy [17].

**Table 1:** Patient index, age, sex, diagnosis used for the study and target volume.

Patient Index	Age at Treatment	Sex	Assigned Diagnosis *	PTV Volume (cm^3^)
1	2	F	Glioma	89.1
2	4	M	Glioma	86.31
3	6	F	Glioma	38.34
4	8	F	Glioma	180.2
5	10	F	Glioma	139.87
6	4	M	Glioma	71.4
7	6	M	Glioma	76.74
8	8	M	Glioma	215.69
9	10	M	Ependymoma	64.38
10	12	F	Ependymoma	97.41
11	13	F	Ependymoma	105.75
12	16	F	Ependymoma	72.57
13	16	F	Ependymoma	95.49

* Note: Diagnoses were assigned for the purposes of this study and were different from the diagnoses listed in the medical records.

Volumetric Modulated Arc Therapy (VMAT) plans were constructed in Phillips’ Pinnacle TPS, while passively scattered and intensity modulate proton plans were constructed in Varian’s Eclipse TPS. A prescription dose of 54 Gy (RBE) in 1.8 Gy (RBE)/fraction over 30 fractions was used for all patients. Proton RBE of 1.1 was used in this study according to ICRU recommendation [18]. Variation of proton RBE was not considered in this study for simplicity, although there are concerns about the correlation between necrosis and the uncertainties of proton RBE [19]. Plans were considered provisionally acceptable when the dose to 95% of the PTV (D_95%_) was 95% (51.3 Gy (RBE)) of the prescription for glioma plans and 90% (48.6 Gy (RBE)) for ependymoma plans. These dose objectives were maintained unless the constraint for the optic chiasm (50 Gy (RBE)) or brainstem (54 Gy (RBE)) was not met. All plans received final approval by a radiation oncologist.

A post planning comparison was performed to assess the congruence between the photon and proton plans. The plan conformity was evaluated in the PTV using the conformity index (CI) as described by Feuvret *et al.* [20]

(1) $$CI=\frac{TV_{RI}}{TV}\times \frac{TV_{RI}}{V_{RI}}$$

where TV is the target volume (PTV), TV_RI_ is the target volume that is covered by the reference isodose, and V_RI_ is the volume of the reference isodose. In this work the reference isodose was 95% (51.3 Gy (RBE)) of the prescription dose. Conformity index has a range from 0 to 1, where 1 is ideal. The Dose to 95% of the PTV was also used to quantify the coverage of the PTV and can be extracted from the cumulative dose volume histograms (DVH) in tabular form.

The dose homogeneity index is a measure of how homogeneous the dose is within the PTV. This metrics was described by Yoon *et al.* [21] and is given by

(2) $$HI=\frac{D_{2\%}-D_{98\%}}{D_{p}}$$

where D_2%_ is the dose to 2% of the PTV volume, D_98%_ is the dose to 98% of the PTV volume, and D_p_ is the prescribed dose. Homogeneity index has an optimal value of 0.

Plans were evaluated on RICN in the brain using the Lyman Kutcher Burman (LKB) probit model for normal tissue complication probability (NTCP) [22]. The ratio of NTCP (rNTCP) was then determined to compare between proton and VMAT plans using the following equation.

(3) $$rNTCP=\frac{NTCP_{proton}}{NTCP_{VMAT}}$$

where the NTCP calculated for protons plans was divided by the NTCP calculated for the VMAT plans.

**Figure 1:**
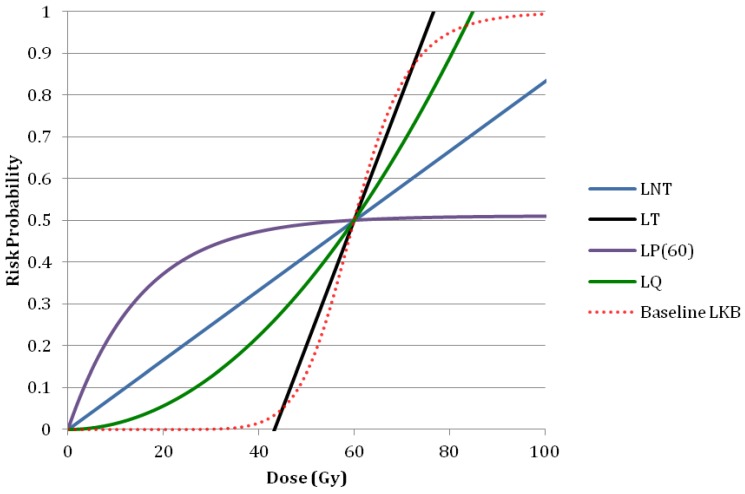
Alternative risk models used to test the sensitivity of rNTCP to the shape of the model used. LNT is the linear no-threshold model, LT is the linear threshold model, LP(60) is the linear plateau model, and LQ is the linear quadratic model.

A sensitivity analysis was used to determine the robustness of treatment plans for comparison. Setup and range uncertainty was simulated using ±1 cm isocenter shifts and ±10% Hounsfield to stopping power calibration curve errors respectively. Additionally sensitivity of the risk model to dose and volume parameter may result in changes in the predicted risk of necrosis. For this reason the predicted risk was computed using a variety of the other risk models and compared to the Lyman Kutcher Burman (LKB) model. These other models included the linear no-threshold model (LNT), linear threshold model (LT), linear plateau model (LP) and linear quadratic model (LQ) and were fit to the same total whole brain dose of 60 Gy (RBE) to induce 50% complication (TD50) as the LKB model [22]. The resulting fit can be seen in Figure 1.

## 3. Results and Discussion

### 3.1. Planning Comparison

**Figure 2:**
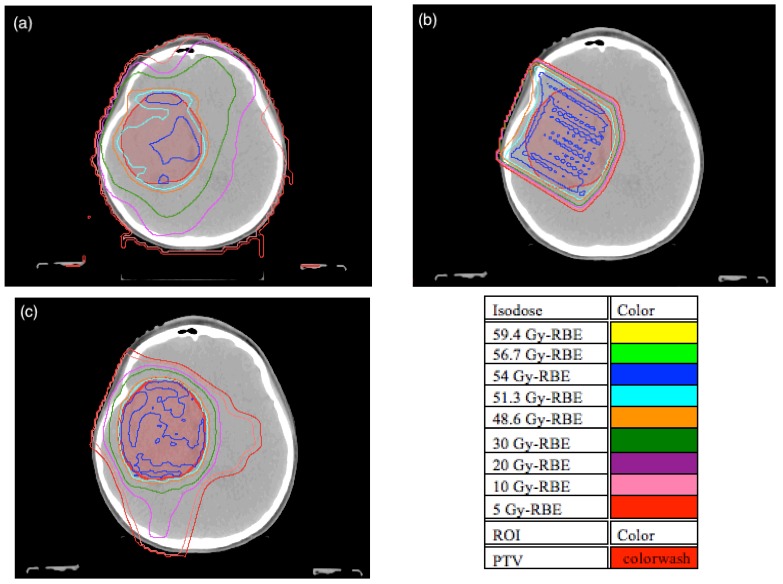
Patient 5 isodose distribution at isocenter CT slice location for (**a**) volumetric modulated arc therapy (VMAT), (**b**) passively scattered proton therapy (PSPT), and (**c**) intensity modulated proton therapy (IMPT).

**Figure 3:**
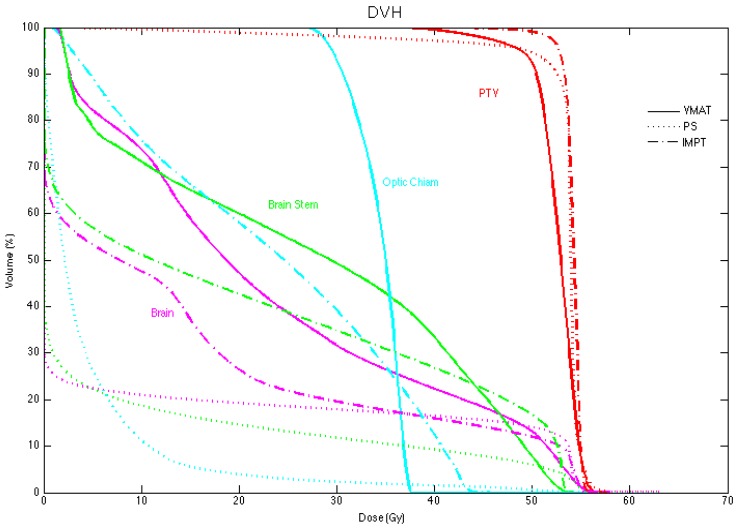
The dose volume histograms (DVH) for patient 5. VMAT plans are represented with the solid lines, PSPT with the dotted lines and IMPT with the dash-dot lines. Different regions of interest are represented with different colored lines.

**Figure 4:**
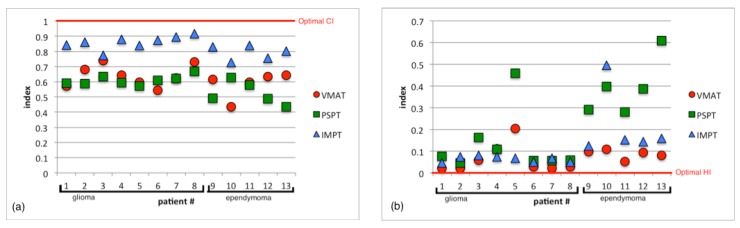
(**a**) The conformity of the dose to the PTV represented by the conformity index (CI) for VMAT, PSPT, and IMPT plans. (**b**) The homogeneity of the dose to the PTV represented by the homogeneity index (HI) for VMAT, PSPT, and IMPT plans.

Patient plans were compared on the basis of different dose metrics for the PTV and whole brain. In the PTV, CI, HI and the dose to 95% of the PTV (D_95%_) were used for comparison. Patient isodose distributions and DVH were also used to visually compare patient plan metrics and they are shown in Figure 2 and Figure 3 for a typical patient. The results for CI and HI are displayed in Figure 4a,b respectively.

PSPT plans showed no significant difference in the conformity compared to VMAT with average CI values of 0.58 ± 0.07 and 0.62 ± 0.08, respectively. The IMPT plans resulted in significantly better conformity than the VMAT plans with an average CI value of 0.83 ± 0.06. Merchant *et al.* [23] also found better conformity over photon therapy when using an active scanning proton technique for a variety of different brain cancer treatments. However the specific photon modality that was used for treatment was not specified in that study. Baumert *et al.* [24] also found increase conformity for IMPT and PSPT for irregularly shaped tumors compared to intensity modulated stereotactic radiotherapy but conformity was comparable between modalities for large concentric treatment volumes.

Both the VMAT and IMPT plans showed good homogeneity with average HI values of 0.07 ± 0.05 and 0.12 ± 0.12. On the other hand, PSPT plans had significantly worse homogeneity than the VMAT plans with an average HI of 0.23 ± 0.19. Contrary to our study, Bolsi *et al.* [25] found that PSPT had better homogeneity than photons. However their comparison was with 3D conformal radiation therapy and not with VMAT, which typically has better conformity. Howell *et al.* [16] also found that photon plans had greater dose heterogeneity than passively scattered proton therapy (PSPT) for medulloblastoma. Again, their study was not comparing PSPT to VMAT and additionally they used D_5%_ and D_95%_ for homogeneity calculation while D_2%_ and D_98%_ were used in our study. Kozak *et al.* [26] did compare protons to intensity modulated radiation therapy (IMRT), which has a similar dose delivery to VMAT and found that homogeneity was comparable between modalities.

PSPT plans resulted in a significantly lower average D_95%_ than the VMAT plans and 5 of the 13 plans did not meet the criteria for acceptable coverage. IMPT and VMAT average D_95%_ values were not significantly different from one another and only one patient from each modality failed to meet the coverage criteria (patients 10 and 5, respectively). It is important to note that although some of these plans failed to meet the planning criteria they were still considered clinically acceptable. The failure may have been a result of the proximity of the treatment volume with respect to the brainstem. For this reason the PTV planning goal was sacrificed in these cases since the constraint on the dose to the brainstem was considered as the paramount objective. Other studies have also found similar coverage between proton and IMRT photon plans but superior coverage for protons when compared against photon conformal radiation therapy [23,25,27].

**Figure 5:**
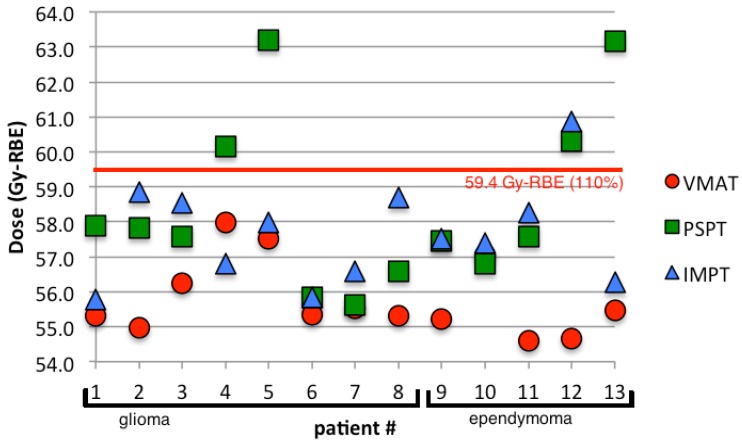
The maximum dose to the brain for VMAT, PSPT, and IMPT plans.

The whole brain was compared on the basis of maximum dose, mean dose and volume of the brain receiving 5, 10, 50, 52, and 56 Gy (RBE). The maximum dose to the brain for all patients is displayed in Figure 5. Both IMPT and PSPT plans resulted in a significantly higher maximum dose to the brain than the VMAT plans with average maximum values of 57.6 ± 1.44, 58.5 ± 2.51, and 55.8 ± 1.08 Gy (RBE), respectively. The mean dose to the brain was significantly lower for both the PSPT and IMPT plans compared to VMAT with average mean values of 7.9 ± 1.1, 10.3 ± 4.0, and 17.5 ± 5.6 Gy (RBE), respectively. The reduction of the mean dose was expected since the protons have been shown to reduce the volume of surrounding tissue irradiated compared to photon therapies, thereby reducing the mean dose. Kozak *et al.* [26] also found this reduction in the mean dose to surrounding normal tissues. The increase in the maximum dose is likely due to tissue heterogeneities [28]. Baumert *et al.* [27] and Kozak *et al.* [26] also found an increase in the maximum dose from protons compared to photon therapies. Additionally the percentage volume of the brain receiving 5 and 10 Gy (RBE) was significantly reduced for PSPT and IMPT plans compared to VMAT while doses greater than 50 Gy (RBE) showed no significant difference between modalities. VMAT plans resulted in a low dose bath of 10 Gy (RBE) to nearly 50% of the brain. PSPT and IMPT reduced the volume receiving 10 Gy (RBE) to nearly half of that from VMAT. Other studies have found similar results of normal tissue sparing for brain treatments [23,25,27,29,30,31].

### 3.2. Normal Tissue Complication Probability

**Figure 6:**
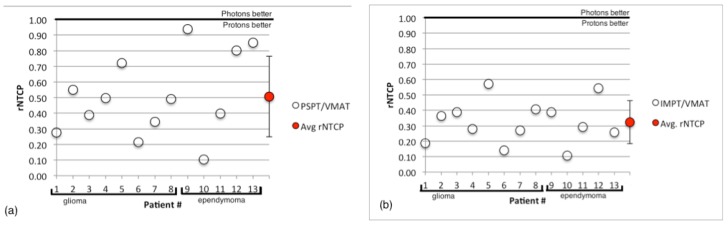
(**a**) The ratio of risk (rNTCP) for PSPT plans compared to VMAT for all patients. (**b**) The ratio of risk (rNTCP) for IMPT plans compared to VMAT for all patients. The average value of rNTCP represented by the red circle with standard deviation error bar.

The values of rNTCP are displayed in Figure 6, with average values of 0.51 ± 0.3 for PSPT compared to VMAT and 0.32 ± 0.1 for IMPT compared to VMAT. rNTCP values were compared to 1 using the Wilcoxon signed rank test and were found to be significantly lower than 1, indicating that both proton modalities should confer a reduction in the risk of necrosis.

### 3.3. Sensitivity Analysis

**Figure 7:**
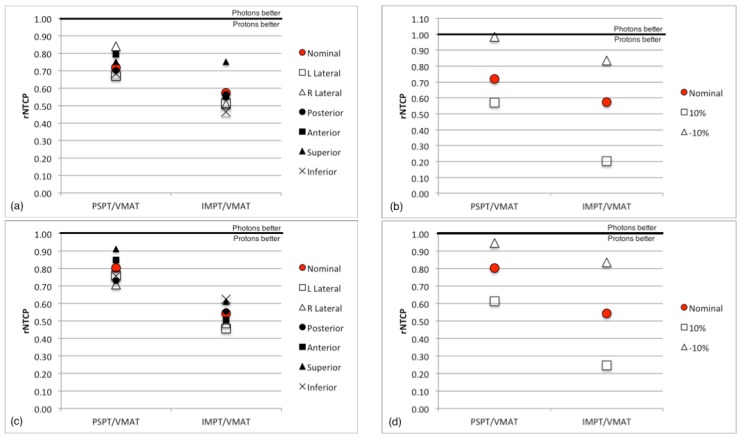
(**a**) The change in rNTCP with isocenter shifts and (**b**) with ±10% calibration curve errors for patient 5. (**c**) The change in rNTCP with isocenter shifts and (**d**) with ±10% calibration curve errors for patient 12.

Isocenter shifts were performed on patients 5 and 12. The result of a 1cm shift on the ratio of predicted risk can be seen in Figure 7a,c respectively. For patient 5 the greatest change in rNTCP was 0.12 from a right lateral shift in the PSPT plan and 0.18 from a superior shift in the IMPT plan. The greatest changes in rNTCP for patient 12 for PSPT and IMPT plans resulted from a superior shift and left lateral shift and were 0.11 and 0.08, respectively. Overall the changes in the dose metric for isocenter shifts were minimal and resulted in minor variations in NTCP and the ratio of NTCP compared to the nominal plans. PSPT plans seemed to be slightly more susceptible to setup error, which is likely due to the single beam arrangement that was used in most of the PSPT plans.

Range errors from shifts in the CT calibration curve resulted in changes to the relative risk of necrosis (rNTCP) across proton planning modalities. An increase of 10% in the calibration curve resulted in reduction of the proton range within the target as well as within the brain and a reduction in the risk. A decrease in the calibration curve would have the opposite effect and an increase in the risk of necrosis was the result. This is in accordance with calibration reports for HU-proton stopping power in the literature [28,32,33,34]. rNTCP results for calibration curve errors for patients 5 and 12 are displayed in in Figure 7b,d, respectively. In both patients an increase in the calibration curve by 10% resulted in a reduction of rNTCP. A reduction of the calibration curve by 10% has the opposite effect and increased rNTCP resulting in a decreased benefit for either proton treatments over VMAT. Even with such a large shift all of the proton NTCP values are still less than or equal to VMAT NTCP resulting in a rNTCP of less than 1 for all range errors.

**Figure 8:**
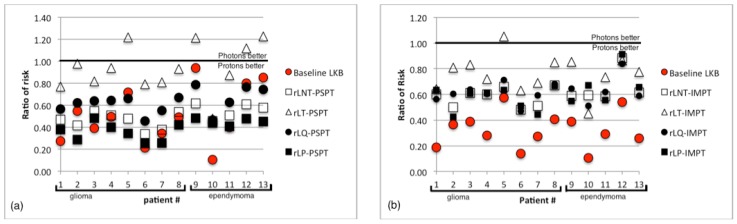
The ratio of risk of necrosis for (**a**) PSPT plans and (**b**) IMPT plans compared to VMAT plans, calculated with baseline Lyman Kutcher Burman (LKB) model, the linear no threshold (LNT), linear threshold (LT), linear quadratic (LQ), and linear plateau (LP) models.

The choice of risk model used can cause large variations in the NTCP results as seen with the alternate risk models chosen in this paper. Most alternative models we considered showed a reduction in the predicted risk when PSPT or IMPT was used instead of VMAT. The ratios of risk from alternative risk model calculations are displayed in Figure 8 for PSPT and IMPT. All models resulted in a ratio of risk significantly less than one with the exception was the PSPT plans calculated using the LT model: The average ratio of risk for these plans was 0.9 ± 0.2 and 4 of the 13 plans had a ratio above 1. The ratio of risk was not significantly less than one for these plans. There was also a single IMPT plan calculated using the LT model that had a ratio of risk above 1, but the average ratio of risk were still found to be significantly less than one. Our findings clearly revealed that LKB, LNT, and LP models were strongly influenced by the volume of the brain irradiated at low dose. And although volumes receiving low dose may increase the chance of developing necrosis we believe that necrosis is likely a deterministic effect with a threshold dose and that the high dose region may strongly impact the initial necrotic incident. Additionally, the linear threshold (LT) model had a threshold at about 43 Gy (RBE) and resulted in absolute risks of necrosis of approximately 1%–5% which better agreed with retrospective studies of similar prescriptions and fractionation and had observed rates of necrosis of approximately 3%–5% [10,11,14].

## 4. Conclusions

The risk of radiation necrosis is of particular concern in pediatric patients with brain cancer because the current cure rates for these cancers are high and life expectancy is long and necrosis is a severe side effect of treatment that is potentially fatal. The findings of this study indicate that choosing either PSPT or IMPT over VMAT when treating some pediatric brain cancer patients can reduce the predicted risk of radiation necrosis. The ratio of risk was sensitive to proton range uncertainty but still showed significant risk reduction for proton plans over VMAT. The choice of risk model had little impact on the ratio of risk for necrosis and the qualitative findings of this study still hold in most cases. A more accurate dose-risk model based might be achievable with the availability of a large sample of radiotherapy patient data on necrosis that takes into account dose, volume, fractionation, and RBE variation.
